# PP54 Towards Equitable Pricing: Unraveling The Complexities Of External Reference Pricing In Medical Devices

**DOI:** 10.1017/S0266462324002253

**Published:** 2025-01-07

**Authors:** Birol Tibet, Ayse Gokcen Kilic, Zahra Ahangar Atashi, Guvenc Kockaya

## Abstract

**Introduction:**

External reference pricing (ERP) serves as a widely adopted method for establishing authorization and reimbursement prices for pharmaceuticals in Europe. However, its application within the medical device (MD) industry remains shrouded in uncertainty. This study seeks to investigate the feasibility of integrating ERP into the realm of MDs, identify pertinent challenges and ambiguities, and propose solutions to bolster affordability and accessibility.

**Methods:**

The methodology encompassed a systematic search across diverse databases (Google Scholar, Google, ResearchGate, PubMed), utilizing combinations of relevant keywords, like “medical device,” “external reference pricing,” “price,” “reimbursement,” “affordability,” “challenge,” and “implement.” Articles were thoroughly evaluated by each researcher independently, followed by a consensus-based approach for final assessment. Rigorous scrutiny of relevance, potential insights, study design, and methodology ensured a comprehensive review, maintaining a high standard of research quality. This meticulous process guaranteed the inclusion of pertinent literature on ERP and MD pricing, enhancing the study’s depth and breadth.

**Results:**

Global ERP application in the MD sector exhibits significant variability in country selection, pricing methods, and adaptations to market changes. Pricing and reimbursement policies across international contexts contribute to this diversity, involving authorization procedures, regulatory oversight, technology appraisals, and state financing limitations. Challenges in establishing equitable pricing frameworks underscore the need for clear guidelines, regulatory empowerment, regular audits, and international collaborations. Transparent methodologies for reference price calculation are essential. Addressing observed shortcomings involves negotiating lower prices, implementing subsidies, promoting generic competition and local production, and tailoring reimbursement policies to treatment needs, enhancing affordability and accessibility on a global scale.

**Conclusions:**

Ambiguities in implementing ERP for MDs underscore the necessity of equitable pricing frameworks. The highlighted multidimensional strategies and consideration of market dynamics are deemed essential for the feasibility of ERP in MDs. These insights emphasize the cruciality of a structured and transparent roadmap for optimizing ERP in the realm of MDs, holding significant potential for ensuring affordability and accessibility.

